# Single-cell gene and isoform expression analysis reveals signatures of ageing in haematopoietic stem and progenitor cells

**DOI:** 10.1038/s42003-023-04936-6

**Published:** 2023-05-24

**Authors:** Laura Mincarelli, Vladimir Uzun, David Wright, Anita Scoones, Stuart A. Rushworth, Wilfried Haerty, Iain C. Macaulay

**Affiliations:** 1 Earlham Institute, Norwich Research Park, Norwich, NR4 7UZ United Kingdom; 2 Norwich Medical School, The University of East Anglia, Norwich Research Park, Norwich, United Kingdom

**Keywords:** Genomic analysis, Haematopoietic stem cells

## Abstract

Single-cell approaches have revealed that the haematopoietic hierarchy is a continuum of differentiation, from stem cell to committed progenitor, marked by changes in gene expression. However, many of these approaches neglect isoform-level information and thus do not capture the extent of alternative splicing within the system. Here, we present an integrated short- and long-read single-cell RNA-seq analysis of haematopoietic stem and progenitor cells. We demonstrate that over half of genes detected in standard short-read single-cell analyses are expressed as multiple, often functionally distinct, isoforms, including many transcription factors and key cytokine receptors. We observe global and HSC-specific changes in gene expression with ageing but limited impact of ageing on isoform usage. Integrating single-cell and cell-type-specific isoform landscape in haematopoiesis thus provides a new reference for comprehensive molecular profiling of heterogeneous tissues, as well as novel insights into transcriptional complexity, cell-type-specific splicing events and consequences of ageing.

## Abstract (web summary)

Single-cell transcriptomics using long-read sequencing achieves both gene and isoform expression in haematopoietic stem and progenitor cells, providing novel insights into the understanding of alternative splicing in cellular differentiation.

## Introduction

Single-cell RNA-seq (scRNA-seq) technologies are now applied to a broad spectrum of biological systems^1^ with particular impact in the study of stem cell and developmental biology^2–4^. With the advance of short-read technologies capable of analysing many thousands of cells in a single experiment, it has become possible to identify cell types and state transitions in complex biological systems. In particular, scRNA-seq has highlighted the continuous nature of haematopoietic hierarchy. Cell types, previously thought of as discrete entities, have been shown to exist in a continuum of states from stem cells to mature progenitors^5^. Investigating the regulatory events that occur in these state transitions and how they change with age is central to the understanding of the regenerative potential of stem cells in health and disease.

Alternative splicing (AS) of mRNA transcripts is a mechanism by which several isoforms can be generated from individual genomic loci, enabling significant increases in transcriptomic and proteomic complexity. AS can affect many aspects of gene expression, including transcript export from the nucleus, transcript stability, and, critically, the production of functionally distinct protein isoforms. AS is thought to occur in at least 62% of multi-exonic genes in mouse^6^ and up to 95% of multi-exonic genes in human^7^. Increasingly, there is an understanding that isoform (co-)expression in tissues and cells can reveal previously unseen complexities in cell signalling responses, as was demonstrated recently for G-protein coupled receptors^8^.

In haematopoiesis, substantial levels of alternative splicing have been observed in sorted populations of stem and progenitor cells^9,10^, but in general, it remains overlooked in transcriptional studies of haematopoiesis, including scRNA-seq studies. Because the vast majority of scRNA-seq studies of haematopoiesis have used 3’ cDNA sequencing, AS events are unlikely to be captured, and thus an entire class of biologically important information about isoform usage is lost. Advances in long-read sequencing technologies have enabled unequivocal detection of AS isoforms^11^, and recently, these approaches have been adapted to explore full-length transcript sequences from single-cell experiments^12,13^. Here, we have applied an integrated approach for parallel short- (Illumina) and long-read (Pacific Biosciences; PacBio) single-cell sequencing of Fluorescence Activated Cell Sorting (FACS) enriched populations, using the 10X Genomics Chromium, to enable comprehensive profiling of cellular diversity, gene expression and alternative splicing events in the mouse haematopoietic system.

**Fig. 1:**
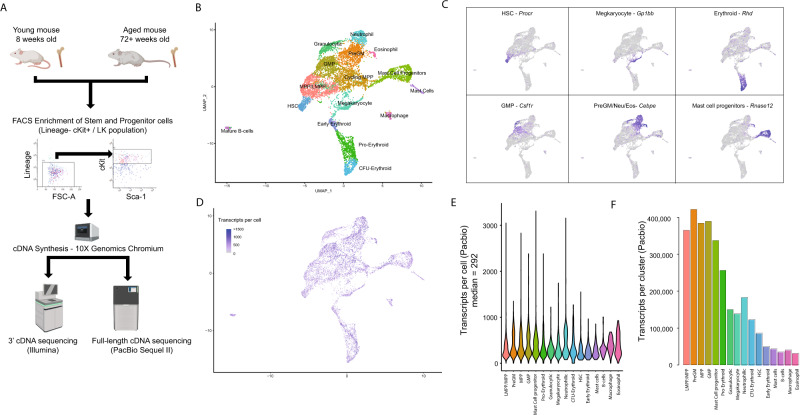
Integrated short- and long-read single-cell RNA-seq of murine haematopoietic stem and progenitor cells. **A** Method overview (created using elements from BioRender) **B** UMAP plot of haematopoietic stem and progenitor cells obtained from 3’ cDNA sequencing of the Lin− cKit+ population. Clusters have been annotated based on known marker gene expression. **C** Selected marker gene expression demonstrating the presence of HSCs, megakaryocytes and myeloid/erythroid progenitors. **D** Numbers of transcripts, derived from long-read sequencing, mapping to individual cells. **E** Distribution of transcripts detected in cells from each of the cell-type clusters. **F** Total number of transcripts detected in each of the cell-type clusters.

We generated cell-barcoded cDNA from haematopoietic stem and progenitor cells isolated from young (8 weeks old) and aged (72+ weeks old) C57BL/6J mice. We then undertook conventional Illumina sequencing of this cDNA to reveal haematopoietic cell states, gene expression, and cell frequency changes associated with ageing. Parallel PacBio sequencing of this full-length cDNA and integration of the cell barcodes annotated the single-cell short-read data with isoform-level information, enabled a survey of isoform expression in the haematopoietic stem cells (HSCs) and their progeny (Fig. 1A). We demonstrate that AS is readily detectable by long-read sequencing of scRNA-seq libraries and that many genes, including known regulators of HSCs and their progeny, are expressed as diverse transcripts, often encoding functionally distinct proteins. These functionally divergent isoforms, undetectable by short-read sequencing alone, indicate that isoform-level analysis is critical for the understanding of cellular systems and states.

## Results

### Annotation of short-read scRNA-seq data with isoform-level information

Using fluorescence-activated cell sorting (FACS), we isolated the Lineage-negative, cKit (Cd117) positive (LK) cell fraction of mouse bone marrow cells, a population containing stem and progenitor cells^14^ from young (8 weeks old, *n* = 3) and aged (72+ weeks old, *n* = 3) mice. We generated standard 10×3’ scRNA-seq libraries from these populations from each mouse, revealing the diversity of cell types present within the 8000 LK cells passing quality control (Fig. 1B).

Analysis of this data identified 15 subclusters within a largely continuous LK population, which could be manually annotated based on classical marker gene expression, including haematopoietic stem cell (HSCs) here associated with *Procr* (*Cd201*) expression^15^, as well as intermediate and committed progenitor cells (Fig. 1B, C, Supplementary Data 1). This includes myeloid, megakaryocytic and erythroid lineages, matching the diversity expected from FACS-based phenotypic analysis of the same population^14^. Small numbers of mature B-cells, myeloid cells and mast cells were also observed but were transcriptionally very distinct from the main stem and progenitor cell cluster and most likely represent low-level contamination with mature cells (Supplementary Fig. 1). In order to examine transcriptional diversity in the haematopoietic system at isoform level, we performed long-read PacBio sequencing (IsoSeq) on each of the six cDNA pools generated from the 10X Genomics platform, similar to an approach recently applied in cerebellar cells^12^. This approach, taking advantage of the cell barcoding technology used in 10X Genomics library preparation, enables isoform identification and association with cell populations and individual cells through the integration of long- and short-read data.

A detailed breakdown of the PacBio sequencing statistics is presented in Supplementary Table 1. In brief, PacBio sequencing yielded a total of 17.9 million circular consensus sequencing (CCS) reads with a median read length of 1471 bases (Supplementary Fig. 2A). These reads mapped to an average of 16,427 genes per sample, representing an average of 33,345 transcripts per sample and an average of 31 reads per transcript. Transcript coverage averaged 74% (Supplementary Fig. 2B), and alternative isoforms were detected for 52.3% of genes (Supplementary Fig. 2C), with the majority of transcripts being protein-coding and spread across a variety of gene categories (Supplementary Fig. 2D, E).

Demultiplexing of the long-reads using the short-read cell barcodes enabled 5.8 million CCS reads (32% of the total) to be assigned to individual cells, reflecting other similar studies^12^ with many reads omitted due to incomplete barcode sequencing/detection in the long-read data, as well as long-reads that could not be assigned due to the QC filtration of the short-read data. Using the multi-modal capabilities of Seurat^16^, we integrated short- and long-read datasets enabling annotation of the short-read dataset with isoform-level transcript expression (Fig. 1D) and allowing side-by-side comparison of gene and isoform expression from the respective platforms. A median of 411 reads, corresponding to a median of 292 transcripts, could be assigned per cell (Supplementary Fig. 3, Fig. 1E), with the number of isoforms detected being too low to allow meaningful comparisons at single-cell resolution. However, with 50,000–500,000 long-reads per cluster, scaling with the number of cells per cluster (Fig. 1F), it is possible to visualise isoform usage across the analysed populations and to associate isoform expression with cell-type clusters.

### Alternative splicing in haematopoietic transcription factor networks

**Fig. 2:**
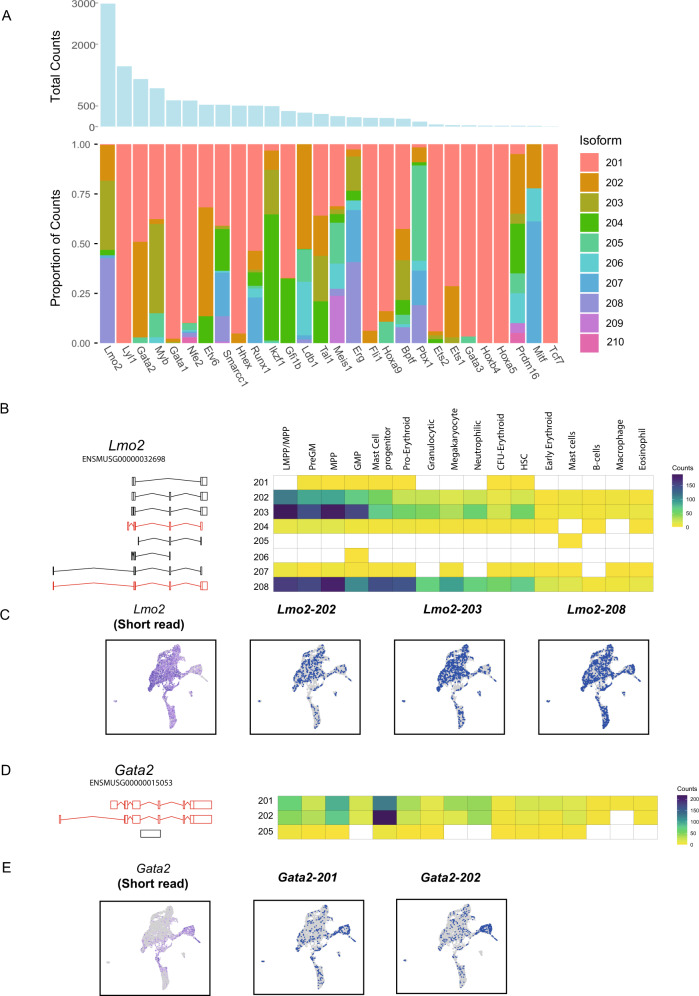
Alternative splicing of haematopoietic transcription factors. **A** Total long-read counts and isoform frequency plot for haematopoietic transcription factors. **B** Cell-type expression of isoforms of *Lmo2*. Transcripts are numbered based on their Ensembl isoform suffix. Transcripts in red encode the same protein sequence, while transcripts in black encode distinct proteins. Transcripts in grey are noncoding. **C** UMAP projection of gene and isoform expression for *Lmo2* in haematopoietic cells. **D** Cell-type expression of isoforms of *Gata2*. Both major transcripts encode the same protein, although the transcript has an alternative 5’ UTR. **E** UMAP projection of gene and isoform expression for Gata2 in haematopoietic cells.

We first screened the dataset to identify AS events in key transcription factors (TFs) that regulate cell fate decisions across haematopoiesis using a regulatory network derived from relevant single-cell studies^17^. Our long-read sequencing detected 28 (of 31) TFs in that network (Fig. 2A), including three predominant isoforms of *Lmo2* - *Lmo2-202*, *Lmo2-203* and *Lmo2-208*, each of which encodes a protein differing in length (228, 220 and 158 amino acids, respectively) with progressive truncation from the N-terminus (Fig. 2B). The major isoform, *Lmo-208* is ubiquitously expressed, while *Lmo2-202* and *-203* show limited expression in the megakaryocyte and erythroid lineages. Additionally, by screening the data for novel exons, we identified a novel, in-frame variant of *Lmo2* with a 297-bp exon supported by 27 long-reads (Supplementary Fig. 4). In human cell lines, long- and short-protein isoforms of *Lmo2* (equivalent to the proteins encoded by *Lmo2-202* and *-203*) have been shown to have distinct functional roles in the formation of TF complexes and regulation of gene expression^18^.

*Gata2* expression, which in our short-read data is restricted to stem and early progenitor cells, mast cell and MkP and erythroid populations, consists of two main isoforms, *Gata2-201* and *Gata2-202* (Fig. 2C). They are translated into the same protein but are differentiated by the usage of distinct distal exons, and have previously been shown to exhibit some lineage-specific expression^19^. Here, both isoforms are most abundant in the mast cell population, with *Gata2-202* showing more restricted expression in this cell type (Fig. 2D, E). We also observe the expression of multiple isoforms of *Ldb1* and *Tal1* (Supplementary Fig. 5A, B) and even in transcripts with relatively low long-read counts, such as *Meis1*, we can observe an exon skipping isoform (exon 11), which encodes a functionally distinct protein, equivalent to *MEIS1D* in human^20^ (Supplementary Fig. 5C).

### Alternative splicing and cytokine receptors

**Fig. 3:**
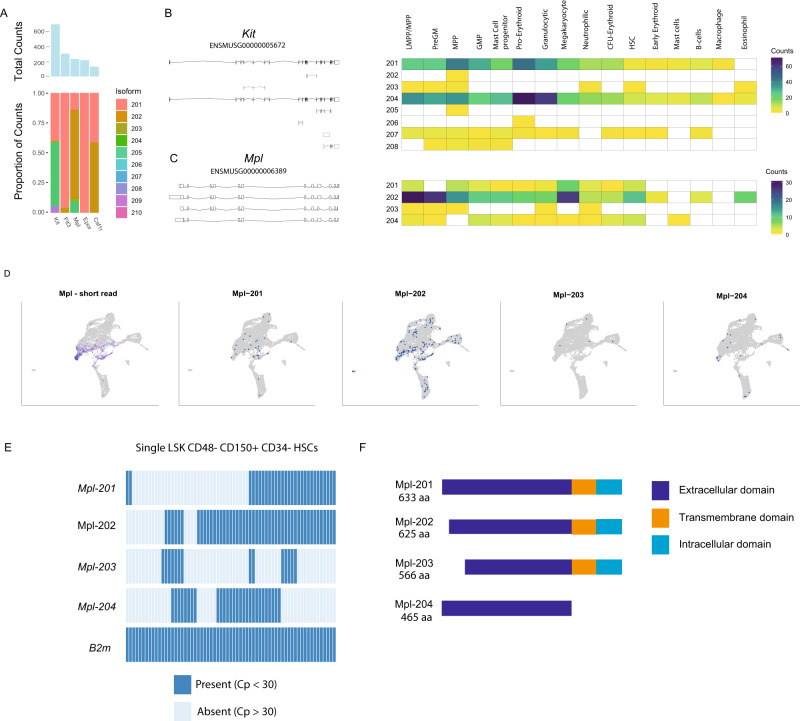
Isoform diversity in haematopoietic cytokine receptors. **A** Total long-read counts and isoform frequency plot for cytokine receptors regulating haematopoiesis. **B** Cell-type expression of isoforms of *Kit*. Transcripts in black encode distinct proteins from the same gene. Those in grey are noncoding. **C** Cell-type expression of isoforms of *Mpl*. Transcripts in black encode distinct proteins from the same gene. Those in grey are noncoding. **D** UMAP projection of gene and isoform expression for Mpl in haematopoietic cells. **E** Detection of *Mpl* isoforms by junction-specific qPCR in individual phenotypic (LSK Cd48− Cd150+ Cd34−) HSCs. Heatmap shows isoform expression in individual cells (columns) of four isoforms of *Mpl*. *B2m* is presented as a positive control. **F** Diagram of the distinct protein structures encoded by the distinct isoforms of *Mpl*.

Transmembrane proteins can determine the cellular response to its environment, particularly regulatory cytokines. We screened the long-read data for isoform expression of a panel of cytokine receptors (Fig. 3A). Two predominant isoforms of the stem cell factor (SCF) receptor *Kit* (*Kit-201* and *Kit-204*) were observed to be expressed in virtually all cell types (Fig. 3B). These isoforms encode proteins differing in length by 4 amino acids (KGNN) which, when absent, results in the loss of a low-complexity region in the juxtamembrane extracellular region of the protein. The human equivalent of these variants has been shown to display distinct signalling activities^21–23^. Four isoforms of *Mpl*, the gene encoding the thrombopoietin (ThPO) receptor^24^, were detectable in both stem cells and megakaryocytes (Fig. 3C, D). The primary isoform, *Mpl-201*, encodes the transmembrane Mpl receptor. *Mpl-202* encodes a shortened version of the protein, lacking eight amino acids in the extracellular domain. *Mpl-204*, which lacks exons 9 and 10 completely, encodes a truncated protein with no transmembrane domain. This isoform is detectable in the stem cell population, and functional studies have indicated that it has an inhibitory role on normal Mpl signalling^24^.

Using junction-targeting qPCR, we examined the distribution of Mpl isoforms in single FACS-isolated HSCs (LSK Cd150+ Cd48− Cd34−) (Fig. 3E). This demonstrated that individual HSCs frequently express more than one isoform, and in some cases, three or four isoforms could be detected in the same single cell. *Mpl-202* is the most common isoform but is frequently co-expressed with *Mpl-204* and also occasionally with *Mpl-203*. The observation that individual HSCs can express more than one transcript encoding distinct protein isoforms (Fig. 3F) of this key cytokine receptor suggests a way in which ThPO signalling could regulate in the functionally heterogeneous or lineage-primed stem cell pool. This highlights the critical need to understand not just gene but also isoform expression in single-cell studies.

### Signatures of ageing

**Fig. 4:**
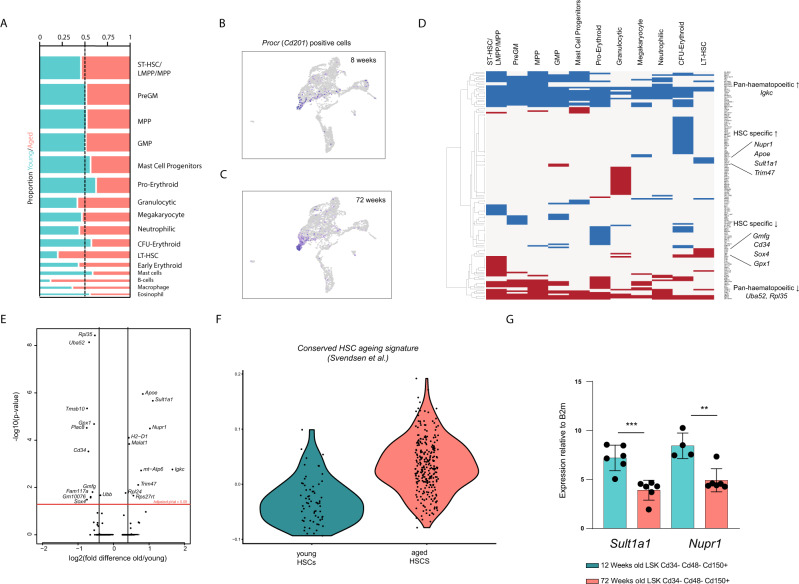
Signatures of ageing in haematopoietic stem and progenitor cells. **A** Frequency of cells per cluster between young and aged mice. **B**, **C** UMAP showing *Procr* expressing cells (HSCs) in young and aged mice, respectively. **D** Differential expression for each cluster, based on short-read data. Data are presented as a heatmap of fold-change values, where blue is upregulated with age and red is downregulated with age. **E** Differential expression in the HSC cluster. **F** Upregulation of aged HSC gene expression signature (from Svendsen et al.^26^) in cells from the aged HSC cluster. **G** qPCR confirmation of *Sult1a1* and *Nupr1* upregulation in individual aged phenotypic (LSK Cd48− Cd150+ Cd34−) HSCs (*** indicates *p*-value < 0.001, ** indicates *p*-value < 0.005).

We integrated short- and long-read data to reveal age-associated signatures in cell type abundance, gene and isoform expression. While the majority of cell type abundances were unchanged between 8- and 72-week-old mice, there was an increased abundance of phenotypic LT-HSCs (Fig. 4A–C) in keeping with previous findings based on stem cells defined by protein marker expression (Lineage negative, Sca-1 positive, cKit positive (LSK) Cd34− Cd48− Cd150+ cells)^25^.

To identify transcriptional signatures associated with ageing across the haematopoietic hierarchy, we performed differential gene expression analysis in each subpopulation using the short-read sequencing data (Fig. 4D). We observed downregulation of genes encoding the cytosolic ribosomal components *Rpl35* and *Uba52* in stem cells and all progenitors and upregulation of the immunoglobulin kappa chain (*Igkc*) throughout the haematopoietic system. To further explore the transcriptional response to ageing, we performed a comparison of the gene expression signature of young and aged HSCs with that from a curated database of HSC ageing genes^26^ showed that the signature from 220 ageing-associated genes was enriched for in our aged HSCs (Fig. 4F) with a general trend of the proportional increase of cells expressing the top 12 most consistent aged HSC marker genes (Supplementary Fig. 6). We observed age-associated upregulation of HSC-specific genes including *Sult1a1* and *Nupr1* in addition to pan-haematopoietic upregulated genes including *Igkc*. We also observed downregulation of *Rpl35*, *Uba52*, *Tmsb10*, *Gpx1*, *Plac8* and *Cd34* (Fig. 4E, Supplementary Data 3). *Sult1a1* and *Nupr1* are highly restricted to the LT-HSC population and are indeed almost exclusively expressed in aged LT-HSCs (Fig. 4E–G), and this increase in expression was confirmed in FACS-purified HSCs (LSK Cd34− Cd48− Cd150+) from young and aged mice by qPCR (Fig. 4G). While *Nupr1* was not detected in the long-read data, the *Sult1a1-203* isoform was the predominant isoform detected, encoding a 188 aa variant of the Sult1a1 protein.

A global comparison of the long-read data from young and aged mice showed an age-associated increase in the expression of noncoding transcripts, including IgV pseudogenes, lncRNAs and transcripts with retained introns (Supplementary Fig. 7). This is consistent with observations that an increased frequency of intron retention has been identified as a signature of ageing in fruitfly, mouse and human^27–29^.

**Fig. 5:**
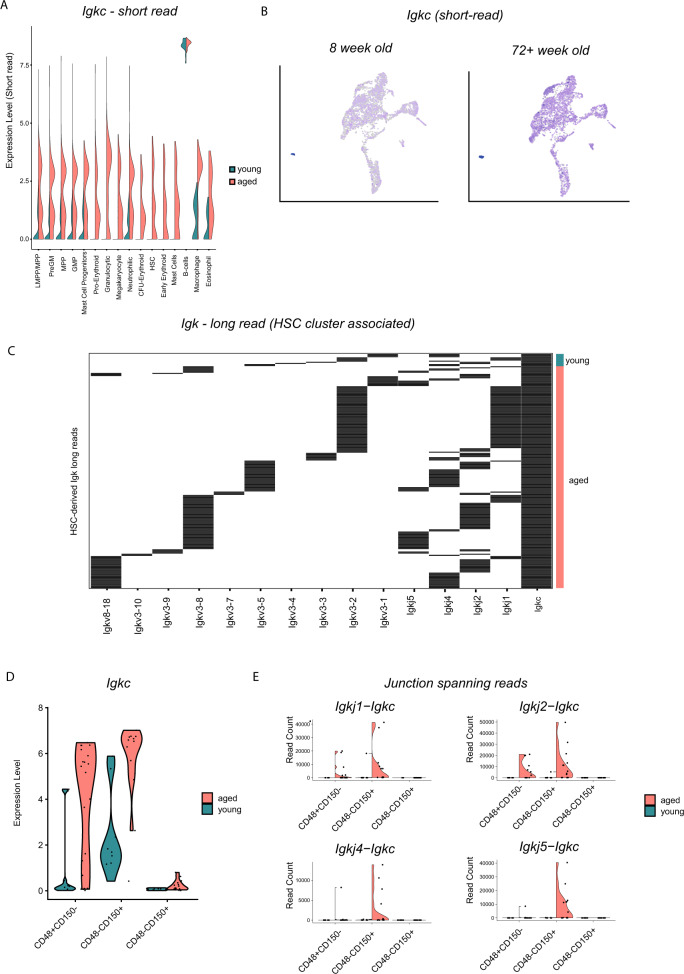
Expression of rearranged Immunoglobulin light chain Kapa (*Igkc*) in aged stem and progenitor cells. **A** *Igkc* expression in short-read profiling of Lin− cKit+ haematopoietic subclusters, split by age. **B** UMAP projection of Igkc expression in short-read profiling of Lin− cKit+ haematopoietic cells, split by age. **C** Observation of full-length, VJ-recombined *Igk* transcripts in long-reads derived from the HSC cluster alone. Data are shown as a heatmap of long-reads (rows) with the presence of V and J regions (columns) indicated in black. **D** Expression of *Igkc* in phenotypic haematopoietic and stem and progenitor cells from young and aged mice. **E** *Igkc-J* region junctions in phenotypic haematopoietic stem and progenitor cells from young and aged mice.

Following the observation that *Igkc* transcripts were upregulated throughout the myeloid progenitor populations and even transcriptionally phenotypic HSCs (Fig. 5A, B), we used the long-read data to determine that these molecules are VJ-recombined *Igkc* transcripts (Fig. 5C, Supplementary Fig. 8), and distinct from IgV pseudogenes (which account for only 1% of immunoglobulin reads sequenced).

To further investigate this, we confirmed the expression of *Igkc* in FACS-isolated pools of LSK Cd48− Cd150+ HSCs, LSK Cd48+ Cd150− cells (predominantly LMPPs) and LSK Cd48+ Cd150+ (early multipotent progenitors) (25 cells per pool) using Smart-seq2 and short-read Illumina sequencing. Here we observed upregulation in cells from aged mice, with slightly increased expression in phenotypic HSCs but more dramatic increases in the progenitor populations (Fig. 5D) with a similar pattern for reads spanning *Igkc* and J-unctions (Fig. 5E), although the detection of junction spanning reads using short-read sequencing is challenging—in contrast to the long-read sequences where the junction detection is unequivocal. A similar pattern was observed for the *Igh* heavy chain (Supplementary Fig. 9C, D). Representative FACS plots for sorted populations are shown in Supplementary Fig. 10.

As non-lymphoid immunoglobulin transcript expression could have arisen from contaminating cells or molecules, we investigated B-cell lineage genes in the short-read data (Supplementary Fig. 11), and Cd45 isoform expression in the long-read data (Supplementary Fig. 11G) confirmed that no B-cell markers were promiscuously expressed throughout the data, nor increase in Cd45r (typically associated with B-cells) detection in cDNA from the aged mice. This indicates that the immunoglobulin molecules detected in these experiments are highly likely to have originated from non-lymphoid committed cells, including myeloid progenitor populations and potentially even transcriptionally phenotypic HSCs.

## Discussion

A complete understanding of transcriptional cellular heterogeneity requires an understanding of the isoform-level expression of genes. The integration of short- and long-read sequencing approaches for single-cell RNA-seq enables the annotation of cells and cell types with isoform-level expression information and thus reveals an additional layer of complexity in cellular transcriptional and functional phenotypes. Here, we integrate these approaches to annotate heterogeneous populations of haematopoietic cells with isoform-level information, revealing that many genes, including key regulators of haematopoiesis, are expressed as multiple—often functionally distinct—isoforms. This approach also enables the integration of previously unannotated isoforms into reference transcriptomes to allow a more accurate annotation of cell-type-specific isoform expression and a better understanding of the contribution of isoform dynamics to cellular function.

Short-read analysis of a typical 10X Genomics experiment measures just 1–3000 genes per cell (~15,000 molecules per cell) representing about 10% of an average cell’s transcriptome (assuming 200,000 mRNA molecules comprising 5–15,000 genes^30^, and thus ~10% of the cell’s transcriptome is “read” in a standard 10X genomics experiment. Our long-read sequencing measures a median of 292 transcripts per cell (from a median of 411 reads per cell), which is approximately 1.5% of the transcripts present within a hypothetical cell. In the course of our and other^12^ analyses of single-cell long-read data, many reads are discarded due to incomplete capture of the cell barcode, and thus, only ~30% of reads can be assigned to single cells, which is a significant limitation of the approach as it currently exists.

We would estimate that without further technical improvement, 100 times more sequencing would reveal the true extent of transcript diversity per cell. This is presently not practical, and methods for more detailed and focussed analysis of smaller numbers of (e.g., FACS isolated) cells are required. In the present case, we use this approach to annotate cell populations with isoform information and to inform single-cell resolution analysis by qPCR of a single gene, but methods which capture global isoform diversity at single-cell resolution would be highly desirable.

With this approach, we demonstrate that over half the genes detected in a standard single-cell analysis of the haematopoietic hierarchy are actually present as multiple isoforms, including transcription factors and transmembrane receptors with key roles in haematopoietic differentiation. Complexes such as Lmo2/Gata2/Ldb1/Tal1 may be readily subject to regulation through isoform expression, with distinct protein isoforms having distinct roles within the overall complex. The finding that many of the transcription factors comprising established networks are present as multiple isoforms suggests an additional layer of complexity that should be taken into account, for example, when building gene regulatory networks from single-cell expression data.

Similarly, it may be that alternative splicing events underlie how phenotypically highly similar cells respond differently to extrinsic signals. We detected multiple isoforms of the transcript encoding the ThPO receptor Mpl, a key regulator of stem cell maintenance and megakaryocyte differentiation, and identified that these isoforms, with functionally distinct protein products, are heterogeneously expressed in phenotypic HSCs. We demonstrate that single HSCs often express more than one isoform of *Mpl*, with each isoform potentially having antagonistic responses to ligand binding. Functional heterogeneity in isoform expression/co-expression could potentially have a critical role in how individual HSCs respond to ThPO, and it would be of considerable interest to understand the role this might play in both steady-state and stress haematopoiesis.

The integrated analysis of short- and long-read data further allowed the identification of multiple signatures of haematopoietic ageing. Here, we have used 7-week-old mice as young and >72-week-old mice as aged, which most likely represents an early rather than an extreme ageing phenotype. Consistent with previous reports, we observe an expansion of a transcriptionally phenotypic HSC population with ageing, while other cell populations remain largely unchanged, and using a compilation of existing studies of HSC ageing, we confirm that the aged cells here express a signature of HSC ageing and that this signature is restricted to HSCs within the LK population. Although *Sult1a1* and *Nupr1* have previously been observed to be upregulated in aged HSCs, we here demonstrate that their expression is exclusive to HSCs and not seen elsewhere in the haematopoietic hierarchy. The genes encoding Nupr1 and Sult1a1 are co-localised in a 50 kb region of chromosome 7, and their elevated expression has previously been shown to be associated with an age-related increase in the H3K4me3 chromatin mark in HSCs^31^. *Sult1a1* encodes for a sulfotransferase which acts on substrates including hormones and neurotransmitters, and *Nupr1* has a regulatory role in cell proliferation and apoptosis, but neither has a described functional role in haematopoiesis^31,32^.

Although there was a clear transcriptional response to ageing, both in HSCs and the wider haematopoietic hierarchy, the usage of isoform was remarkably stable at the resolution analysed here. We observed no specific changes in isoform expression between young and aged mice but the long-read data did show marginally increased expression of lncRNAs. Highly tissue-specific changes in lncRNA expression in aged human tissues have been reported^33^, and abnormally elevated expression of some lncRNAs seems to relate to telomere shortening and senescence^34^. Similarly, an overall increase in intron retention was observed; however, neither signature was specific to any particular gene or lncRNA, indicative of global dysregulation rather than a specific functional response.

The combined use of short- and long-read sequencing enabled the detection of *Igkc* upregulation in aged haematopoietic cells, including transcriptionally phenotypic myeloid progenitors and even HSCs. This was highly unexpected but perhaps not without precedent. Previous work has shown that unrecombined *Igkc* transcripts are expressed in aged LT-HSCs possibly as a result of epigenetic dysregulation^35^, and a recent scRNA-seq study of HSC ageing also detected *Igkc* as the most upregulated transcript with ageing^36^ (Supplementary Fig. 12A). Interestingly, a reanalysis of the Chambers et al.^35^. data in the ageing database described in Svendsen et al.^26^. showed that many *Igkv* loci were upregulated in aged HSCs (Supplementary Fig. 12B), and *Igkc* and *Igv1* expression was also observed to be upregulated in microarray analysis of gene expression in vWF+ platelet-biased HSCs when compared with vWF- HSCs^37^ (Supplementary Fig. 12C). Thus, there is precedent for the aberrant expression of Igkc in ageing HSCs.

The expression of these transcripts is dependent on genomic rearrangements, typically seen in committed lymphoid progenitors downstream of HSCs, but there is an increasing body of evidence that the expression of recombined Ig molecules in non-lymphoid cells is possible^38–40^, including human CD34+ cord blood stem and progenitor cells^41^ and acute myeloid leukaemia^42^. Indeed, the expression of IgG has been confirmed at both the transcriptional and protein levels in human epithelial cancer cells^43–46^, with a restricted variable region repertoire which exerts profound pro-tumorigenic effects^47^.

Further studies of the causative or associated genomic and epigenetic events underlying this aberrant expression will be essential to confirm the genomic origin of immunoglobulin expression in non-lymphoid cell types, including multipotent stem and progenitor cell populations. Currently available methods to sequence immunoglobulin recombination at the genome level regard “low input” as 100,000–200,000 cells (Chovanec et al.^48^), making rare cell-type specific measurements immensely challenging. At present, methods that enable high-confidence single-cell *genomic* recombination analysis—using targeted, high-fidelity amplification and sequencing—have not been described. These approaches, ideally coupled with capture of the cell’s functional or transcriptional phenotype, will be essential to explore the origins of non-lymphoid recombinant immunoglobulin expression and, if confirmed, address the mechanisms by which expression of these recombinant molecules occurs.

Overall, our study not only provides a comprehensive picture of gene and isoform expression in a variety of haematopoietic cell types but also offers novel insights into transcriptional signatures of ageing. Our results highlight the need to further characterise isoform diversity at the single-cell level and to build an isoform atlas for different cell types to reveal the full extent of transcriptional heterogeneity in development, ageing and disease. With continued improvements in the throughput of long-read sequencing library preparation and platforms and the development of methods targeting specific cells or transcripts, we envision that long-read sequencing will enable detailed characterisation of the total landscape of isoform diversity at the single-cell level at a scale comparable to current short-read based methods. This will be applicable to large-scale atlassing studies of cellular heterogeneity and in haematological malignancies where mutations in splicing factors are common^49^. Furthermore, advances in mass spectrometry-based single-cell proteomic analysis^50^, combined with long-read transcript discovery, will become a critical tool enabling the confirmation of protein isoform expression.

## Methods

### Stem and progenitor cell isolation

All animal work in this study was carried out in accordance with regulations set by the United Kingdom Home Office and the Animal Scientific Procedures Act of 1986. Bone marrow was isolated from the spine, femora, tibiae, and ilia of 8 weeks and 72 weeks old C57BL/6J mice. Red blood cell depletion was performed with ammonium chloride lysis (STEMCELL Technologies), and lineage-negative cells were isolated using the EasySep Mouse Hematopoietic Progenitor Cell Isolation Kit (STEMCELL Technologies).

The lineage-depleted cells were stained with the following fluorophore-conjugated monoclonal antibodies: Cd105-PE, clone MJ7/18, Miltenyi; Cd4-Vioblue, clone REA604, Miltenyi; Cd11b-Vioblue, clone REA592, Miltenyi; Cd117-Pe Vio770, clone REA791, Miltenyi; Cd8a-Vioblue, clone 53-6.7, Miltenyi; Cd50-Vioblue, clone REA421, Miltenyi; Cd45R-Vioblue, clone REA755, Miltenyi; GR1-Vioblue, clone REA810, Miltenyi; Sca-APC, clone REA422, Miltenyi; Cd48-APC Cy7, clone HM48-1, Miltenyi; Cd150-BV510, clone TC15-12F12, Cd34-PeCy5, MEC147, Miltenyi. Approximately 10,000 LK (Lin−, Cd117+) cells per sample were sorted using the BD FACSMelody cell sorter (BD Biosciences, San Jose, California) into 1× PBS containing 4% BSA. For low-input and single-cell qPCR, a pool of 25 cells and single LSK Cd150+ Cd48− Cd34− HSCs respectively were sorted directly into Smart-seq2 lysis buffer^51^.

### Sequencing of single-cell cDNA libraries

Sorted cells were processed by 3’ end single-cell RNA-Seq using the 10X Genomics Chromium (V2 Kit) according to the manufacturer’s protocol (10X Genomics, Pleasanton, CA) with an increase to 16 cycles for the cDNA PCR amplification. Six libraries were prepared, each from cells from an individual mouse—three aged and three young. Libraries were sequenced on a NextSeq 500 or NovaSeq 6000 (Illumina, San Diego) in paired-end, single index mode as per the 10X Genomics recommended metrics.

Raw Illumina sequencing data were analysed with the 10X Genomics CellRanger pipeline (version 3.0.2) to obtain a single-cell expression matrix object. Subsequent analysis was performed in R using Seurat version 3^16^. Cells showing gene counts lower than 1000 and a mitochondrial gene expression percentage higher than 5% were excluded from further analysis. Within Seurat, data were normalised using NormalizeData (normalisation.method = “LogNormalize”, scale.factor = 10000) and data from multiple samples were merged using the FindIntegrationAnchors and IntegrateData commands.

### Pacific Biosciences Sequel sequencing of single-cell cDNA libraries

Libraries compatible with the Pacific Biosciences Sequel/Sequel II systems were prepared from 800 ng input cDNA generated from each of the six individual mice following the “no size selection” Iso-Seq library preparation method according to the manufacturer’s instructions (IsoSeq Template Preparation for Sequel System V05), with the following modifications: the elution incubation time during AMPure beads purification was increased to 10 min and the second AMPure bead purification step, following the exonuclease reaction, was omitted to optimise library concentration. In total, six libraries were sequenced, each originating from the LK population of an individual mouse.

### Pacific Biosciences long-read analysis

Circular Consensus reads (CCS) were generated using the following parameters: maximum subread length 20,000, minimum subread length 50 and minimum number of passes 3. Reads with identified polydA or polydT were demultiplexed using bbduk https://sourceforge.net/projects/bbmap/) (k = 16, hdist = 3) using the 10X genomics barcodes identified from the short-read analysis. Long reads were mapped to the mouse genome (mm10) using Minimap 2 (v2.17) and to the gencode (vM19) transcriptome.

Novel exons were identified by investigating the alignment of the reads to the transcriptome identifying inserts of at least 21 nucleotides located at exon junctions. To confirm the existence of these exons, the alignment of the reads to the genome was parsed, with exonic sequences located within the previously identified intron and supported by at least two reads retained for further analysis. We further removed any exonic regions overlapping RefSeq annotations (GRCm38, last accessed February 19 2019). To identify reads supporting V(D)J recombination events, we used IgBlast v1.14.0^52^) using default parameters (-min_V_length 9 -min_J_length 0 -min_D_match 5 -D_penalty -2 -J_penalty -2).

### Custom transcriptome annotation

We took advantage of the long-read pacbio data to annotate and explore alternative splicing events using TALON^53^. We identified a total of 11,013 novel transcripts supported by at least five reads and identified in three or more of the samples (44,993 novel transcripts if at least two reads and two samples). Those annotations also enabled the identification of 910 novel cassette exons, and 4118 and 3465 novel alternative 5’ and 3’ splicing sites, respectively. We also identified 143 novel junctions between previously annotated splice sites. We investigated the impact of the novel splicing events on the coding potential of the transcripts. Among the 10,576 novel transcripts arising from protein-coding genes, a total of 6747 transcripts were identified as coding, whereas 3830 were deemed noncoding (Supplementary Data 2).

TALON v.5.0 was used to construct a custom transcriptome annotation. First, 11 genome-aligned sam files were passed to TranscriptClean v.2.0.2 [2] for correction of read microindels (<5 bp) and mismatches, though any non-canonical splice junctions were retained for downstream analyses, and clean-up was not variant-aware. Internal priming artefacts are a known issue with oligo-dT selection methods [3], and this was assessed using a T-window size of 20 bp (equivalent to the primer T sequence), with reads labelled accordingly using TALON functions. Read annotation was performed using the *Mus musculus* Gencode v.M24 reference annotation gtf and GRCm38.p6 genome with minimum alignment identity = 0.9 and coverage = 0.8 on the samples. Identified transcripts were subsequently filtered using a minimum count threshold of *N* = 5 reads in K = 3 samples. Thresholds were selected to balance sensitivity with accuracy. An updated annotation was produced using this filtered set of transcripts. Novel antisense transcripts that perfectly matched existing gene models were removed as unreliable mappings. The TALON custom gtf contains only features detected with reads present in the dataset, so a complete custom transcriptome annotation was compiled by merging the Gencode v.M24 reference and custom TALON gtfs. Transcript coding potential was assessed through frame preservation and applying CPAT2.0^54^ using Gencode long noncoding RNAs (https://www.gencodegenes.org/) and CDS as training sets.

### Data integration in Seurat

In order to reduce the batch effect, count matrices produced by short-read sequencing for individual libraries were combined in Seurat using FindIntegrationAnchors and IntegrateData functions (dims = 1:20). Illumina and PacBio reads were integrated into Seurat using the CreateAssayObject command to add the long-read data to an existing Seurat object already containing the short-read data. This links the demultiplexed long-reads with the short-read data through the cell barcodes present in both.

### Single-cell and low-input RNA-seq and qPCR

Amplified cDNA was generated from sorted cells using the Smart-seq2 protocol^45^. This material was then used as input for qPCR reactions using assays targeting *Sult1a1* and *Nupr1*. Low-volume qPCR reactions were set up using the Mosquito HV instrument (STP Labtech) and analysed using a LightCycler (Roche). For relative abundance, data are presented as expression relative to a housekeeping gene. For single-cell isoform junction detection PCRs, reactions were performed as above using junction-spanning primers. Data are presented as a presence/absence heatmap, where analyses with Ct values < 30 were considered to be expressed.

For low-input RNA-seq, sequencing libraries were prepared from cDNA using the Nextera protocol (Illumina) at a reduced volume using the Mosquito HV instrument (STP Labtech). Libraries were pooled and sequenced on an Illumina NovaSeq 6000 SP Lane.

### Statistics and reproducibility

Single-cell long-read experiments were carried out on samples from each of three young (8 weeks) and three aged (72+ weeks) mice. qPCR data were generated from samples from six individual young and six individual aged mice. Data are presented as expression relative to *B2m* in the same sample (−1/ΔCt), and statistical significance was measured using an unpaired *t*-test comparing normalised expression levels in young and aged stem cells. Low-input RNA-seq data (for immunoglobulin junction detection) were generated from three young and five aged mice.

### Reporting summary

Further information on research design is available in the Nature Portfolio Reporting Summary linked to this article.

## Supplementary information

Supplementary Figures and Data

Description of Additional Supplementary Files

Supplementary Data 1

Supplementary Data 3

Supplementary Data 2

Supplementary Data 4

Reporting Summary

## Data Availability

Sequencing data can be accessed on the NCBI-GEO archive, accession number GSE166709. Source data for the graphs and charts are given in Supplementary Data 4, and any remaining information can be obtained from the corresponding authors upon reasonable request.
