# Supplementary material for: Single-cell gene and isoform expression analysis reveals signatures of ageing in haematopoietic stem and progenitor cells
Source: Commun Biol. 2023 May 24;6:558. doi: 10.1038/s42003-023-04936-6 (PMC10209181; doi:10.1038/s42003-023-04936-6)
Supplement: Supplementary file 1 — Supplementary Figures and Data [file 42003_2023_4936_MOESM1_ESM.pdf]

Supplementary Figures:

Supplementary Figure 1: Marker genes for the 16 identified clusters in Figure 1B. Cluster 1: MPP/LMPP; Cluster 2 PreGM; Cluster 3 MPP; Cluster 4 GMP; Cluster 5 Mast Cell; Cluster 6 Pro-erythroid; Cluster 7 Granulocytic; Cluster 8 Megakaryocyte; Cluster 9 Neutrophilic; Cluster 10 CFU-erythroid; Cluster 11 HSC; Cluster 12 Early erythroid; Cluster 13 Mast cells; Cluster 14 B-cells; Cluster 15 Macrophage; Cluster 16 Eosinophils.

Supplementary Figure 2: Overview of long-read sequencing and isoform detection in scRNA-seq libraries. A) Read length distribution from long-read sequencing of 10X single-cell libraries. B) transcript coverage from long-read sequencing of 10X single-cell libraries. C) Number of isoforms detected per gene. D) TAPPAS classification of detected transcripts by type. E) Distribution of gene and isoform expression within individual gene ontology categories. Genes are categorised on the number of isoforms observed (shown by the colour of the datapoint) and the enrichment of AS events in particular categories is shown by the size of the datapoint.

Supplementary Figure 3: Long read counts A) per cell, shown per cluster and B) total long read count per cluster.

Supplementary Figure 4: Novel exon discovery in *Lmo2*. Representative Pacbio reads are shown above the Ensembl annotated transcripts for *Lmo2*. Multiple novel in frame exons were observed in the long-read data, indicated by arrows, including two which generate isoforms of the transcript which would encode a distinct protein sequence (highlighted in red).

Supplementary Figure 5: Isoform-level expression of hematopoietic transcription factors. Cell-type expression of isoforms of A) *Ldb1* and B) *Tal1*. Transcripts colored red or blue indicate sets of transcripts which encode the same protein, those in black encode distinct proteins from the same gene. C) Sashimi plot of *Meis1* isoforms present in the long read data. Two predominant isoforms encoding distinct proteins were detected, *Meis1-201* and *Meis1-209*, corresponding to the human *MEIS1B* and *MEIS1D* isoforms.

Supplementary Figure 6: Expression of ageing signature marker genes in hematopoietic stem and progenitor cells. A) Expression of a signature of 220 HSC ageing related genes from Svendssen et al., in each sub-cluster of the Lin- cKit+ cell fraction B) Proportional

expression of the most conserved ageing signature markers from Svendssen et al. in cells from the young and aged HSC clusters.

Supplementary Figure 7: Globally differentially expressed gene categories between young and aged long-read datasets. Statistical comparison within gene categories between young and aged long-read datasets were performed on the normalised read counts using a Kruskal-Wallis test.

Supplementary Figure 8: Recombined *Igkc* transcripts detected HSCs using long-read sequencing. IGV plot of the Igkc locus showing reads from aged (top panel) and young (bottom panel) HSC cluster cells only. Full length reads spanning constant, J and V regions were observed in both young and aged mice but with much greater abundance in the aged mice.

Supplementary Figure 9: Expression of rearranged Immunoglobulin heavy chain H (*Igh*) in aged stem and progenitor cells. A) UMAP projection of global *Igh* expression in the Lin cKit+ population. B) Observation of full length, VDJ recombined *Igh* transcripts in long reads derived from the HSC cluster only. Data are shown as a heatmap of long-reads (rows) with presence of V and J regions (columns) indicated in black. C) expression of *Igh* in phenotypic hematopoietic and stem and progenitor cells upon ageing. D) Observation of *Igh-J* junction spanning reads in phenotypic hematopoietic and stem and progenitor cells.

Supplementary Figure 10: Gating strategy and population frequency in young and aged mice. Representative FACS profiles for HSC and progenitor sorting from A) young and B) aged mice**.** Upper panels show the sorting gates, lower panels show backgating for the HSC population C) population frequencies for HSC and progenitor populations (presented as percentage of LSK) in young and aged mice.

Supplementary Figure 11: B-cell lineage marker genes and Cd45 isoform expression in Lin-cKit+ stem and progenitor cells. A-F) Cell type expression of a panel of lymphoid/B-cell lineage marker genes in the lin- cKit+ hematopoietic stem and progenitor cell compartment. B) Representative Pacbio reads are shown above the Ensembl annotated transcripts for the Cd45 locus, Cd45R and Cd45RO are highlighted.

Supplementary Figure 12: Immunoglobulin detection in HSCs from other studies.

Reanalysis of supplementary data from RNA-seq of aged HSCs in A) Herault et al. B) Chambers et al., reanalysed in Svendsen et al. and C) microarray data from vWF-EGFP+ HSCs (Sanjuan-Pla et al. 2013) demonstrate previous detection of Igkc and V regions in gene expression profiling of HSCs.

Supplementary Tables

Supplementary Table 1:

Long-read sequencing statistics (relating to Figure 2)

Supplementary Data

Supplementary Data 1: Marker genes for clusters (relating to Figure 1B)

Supplementary Data 2: Novel exons detected in long-read sequencing data.

Supplementary Data 3: Differential gene expression between young and aged HSCs (relating to Figure 4E)

Supplementary Data 4: Numerical source data (relating to Figures 1-5)

Supplementary Figure 1

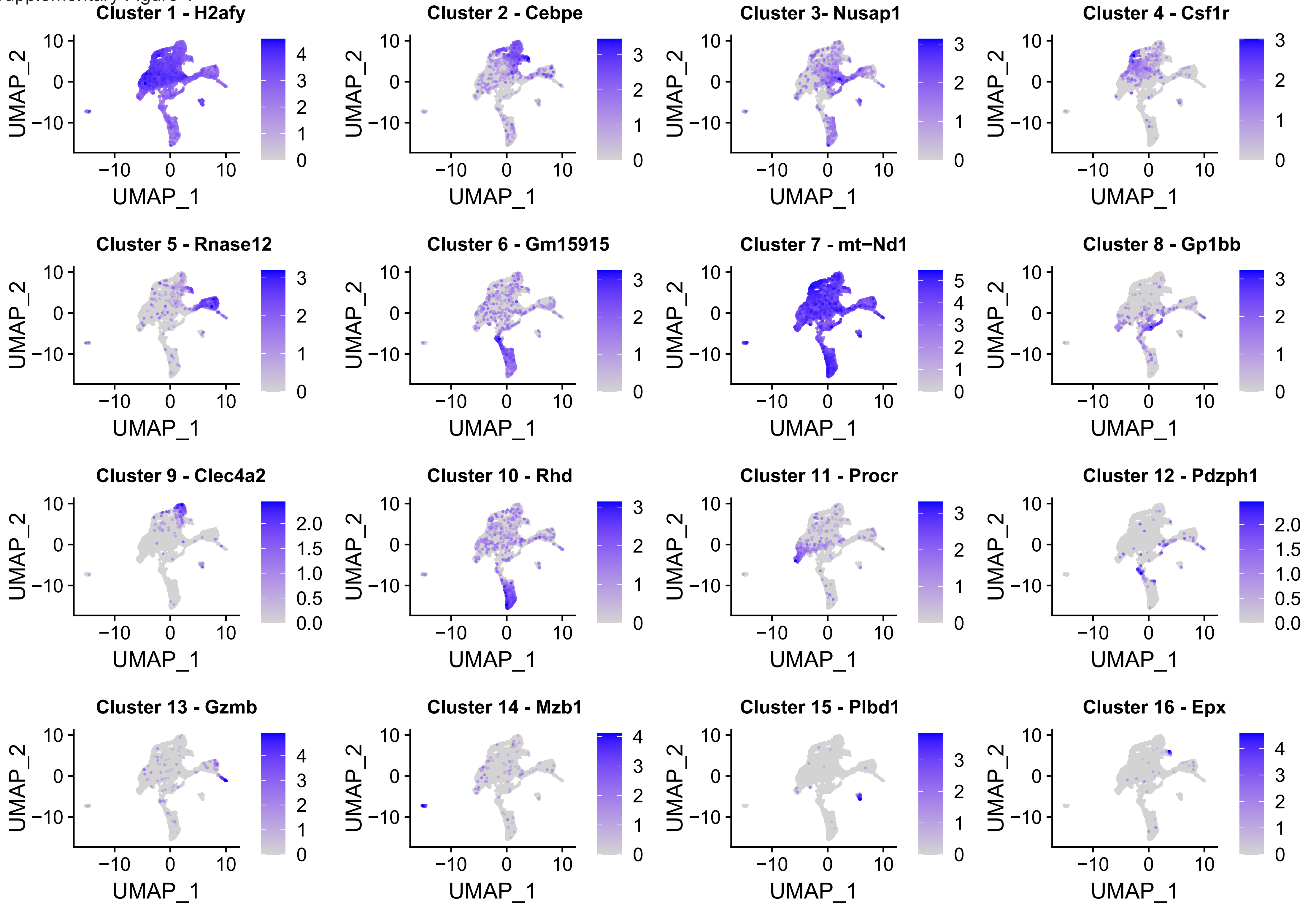

Supplementary Figure 2

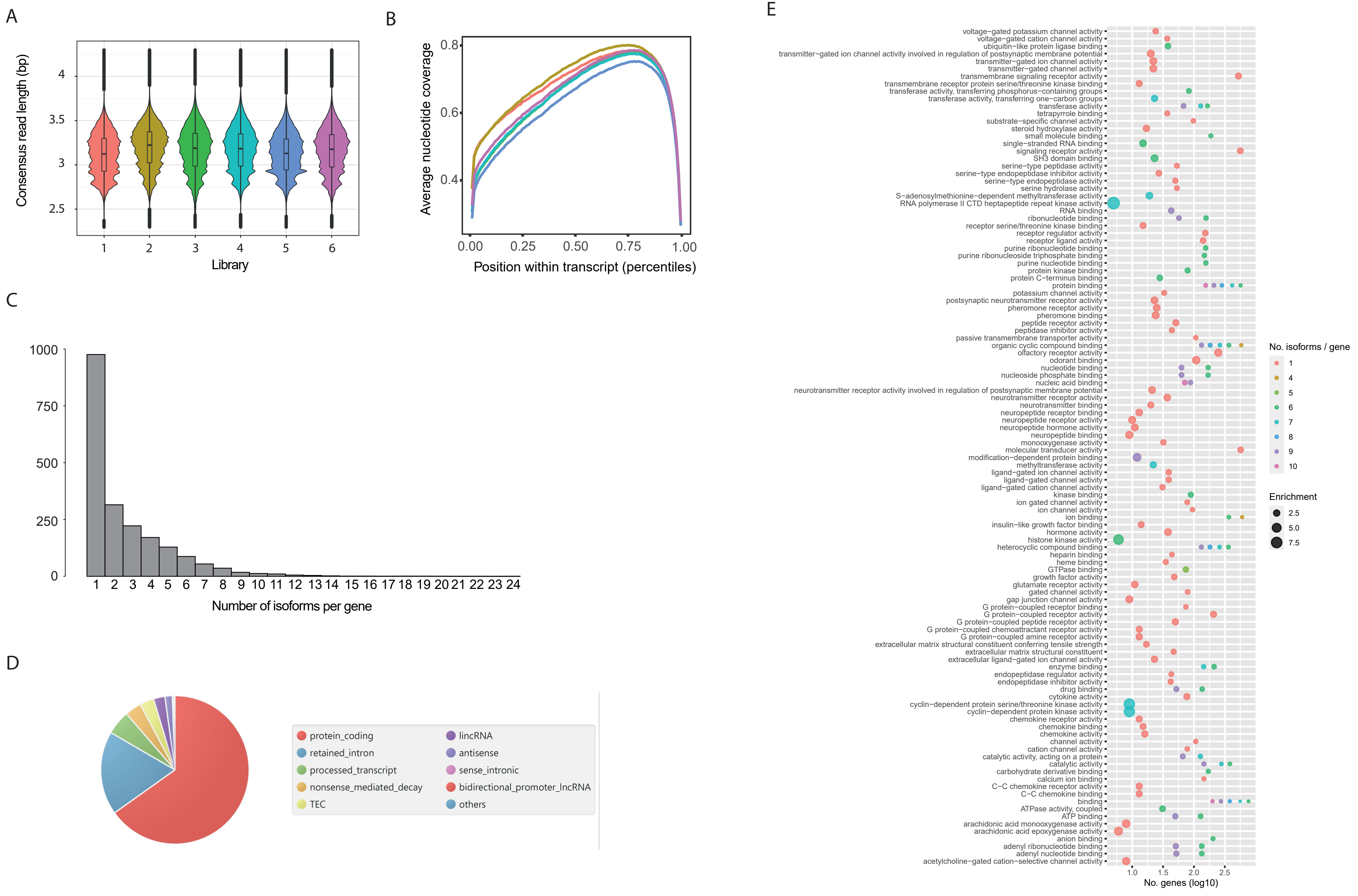

Supplementary Figure 3

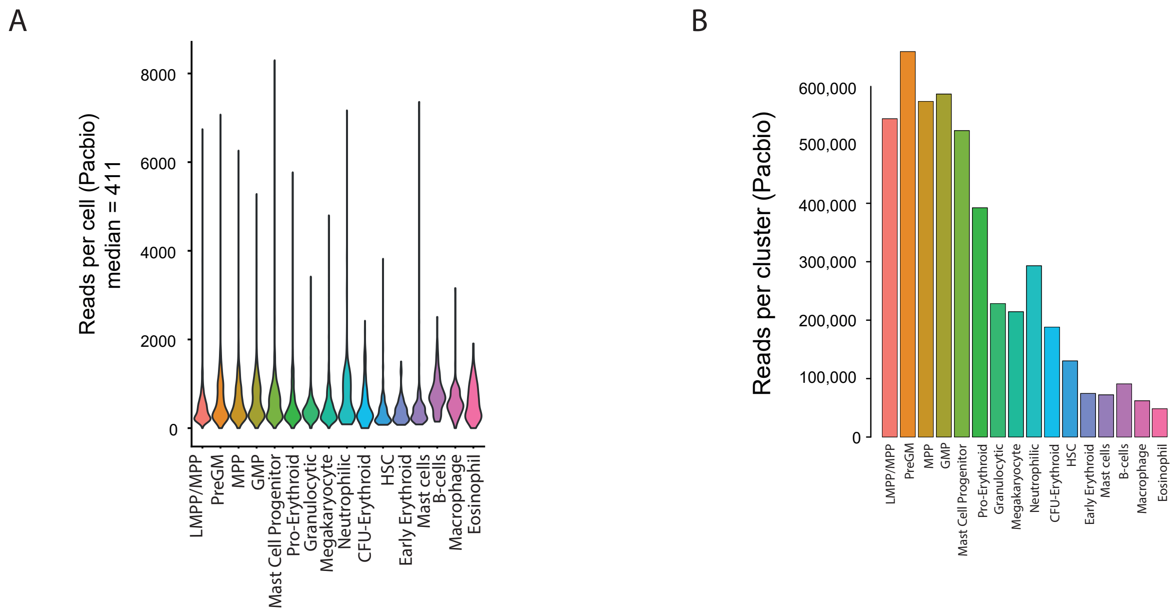

Supplementary Figure 4

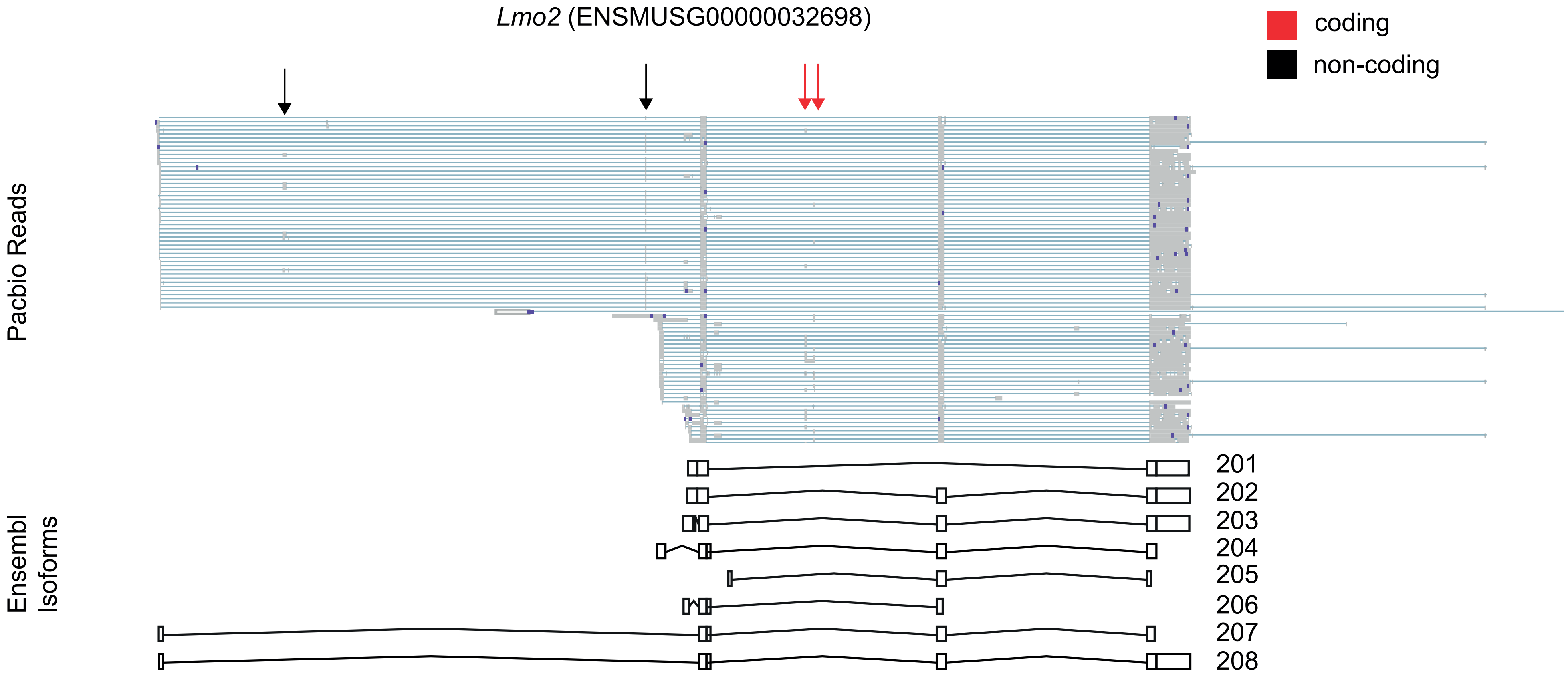

Supplementary Figure 5

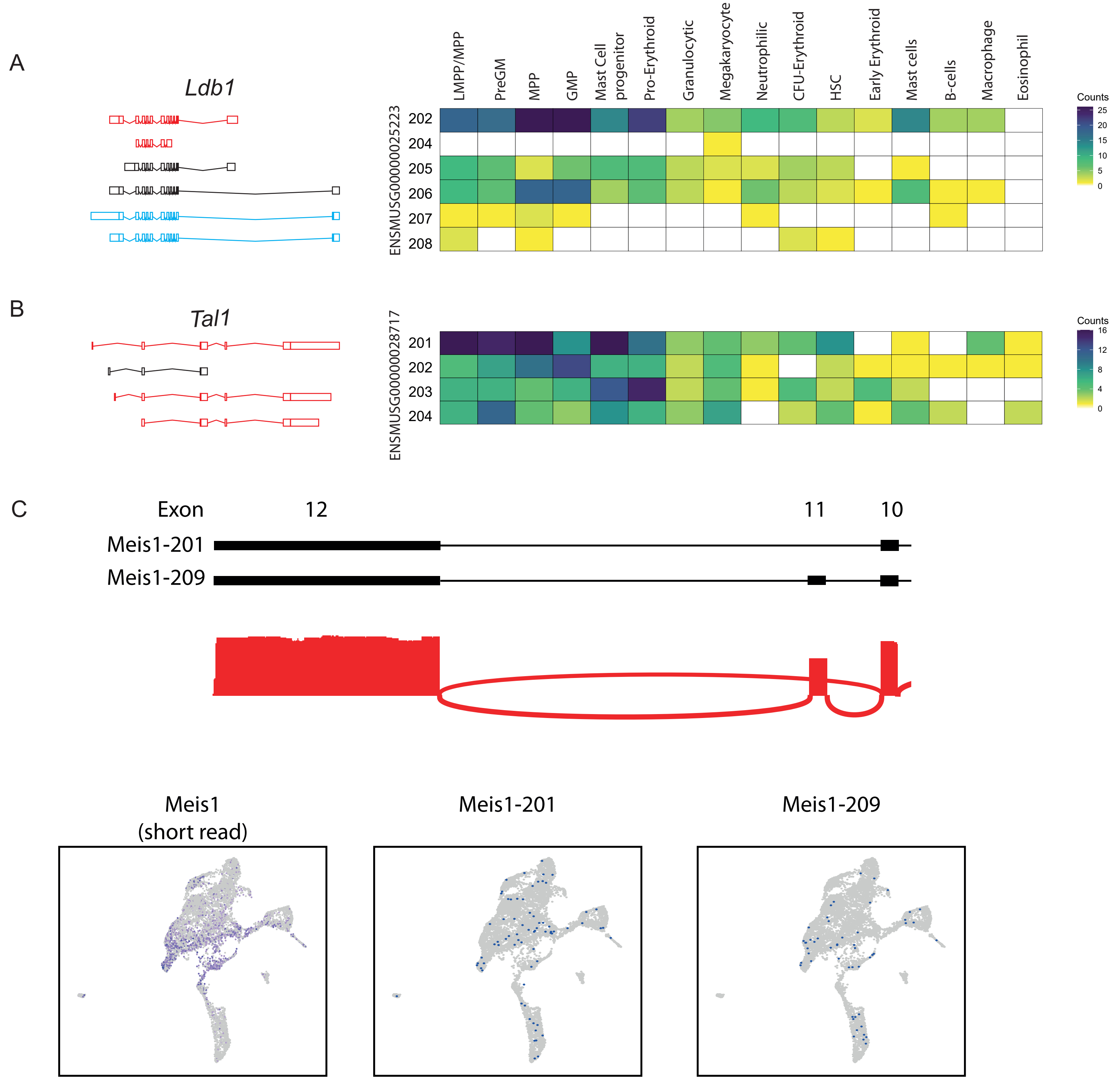

Supplementary Figure 6

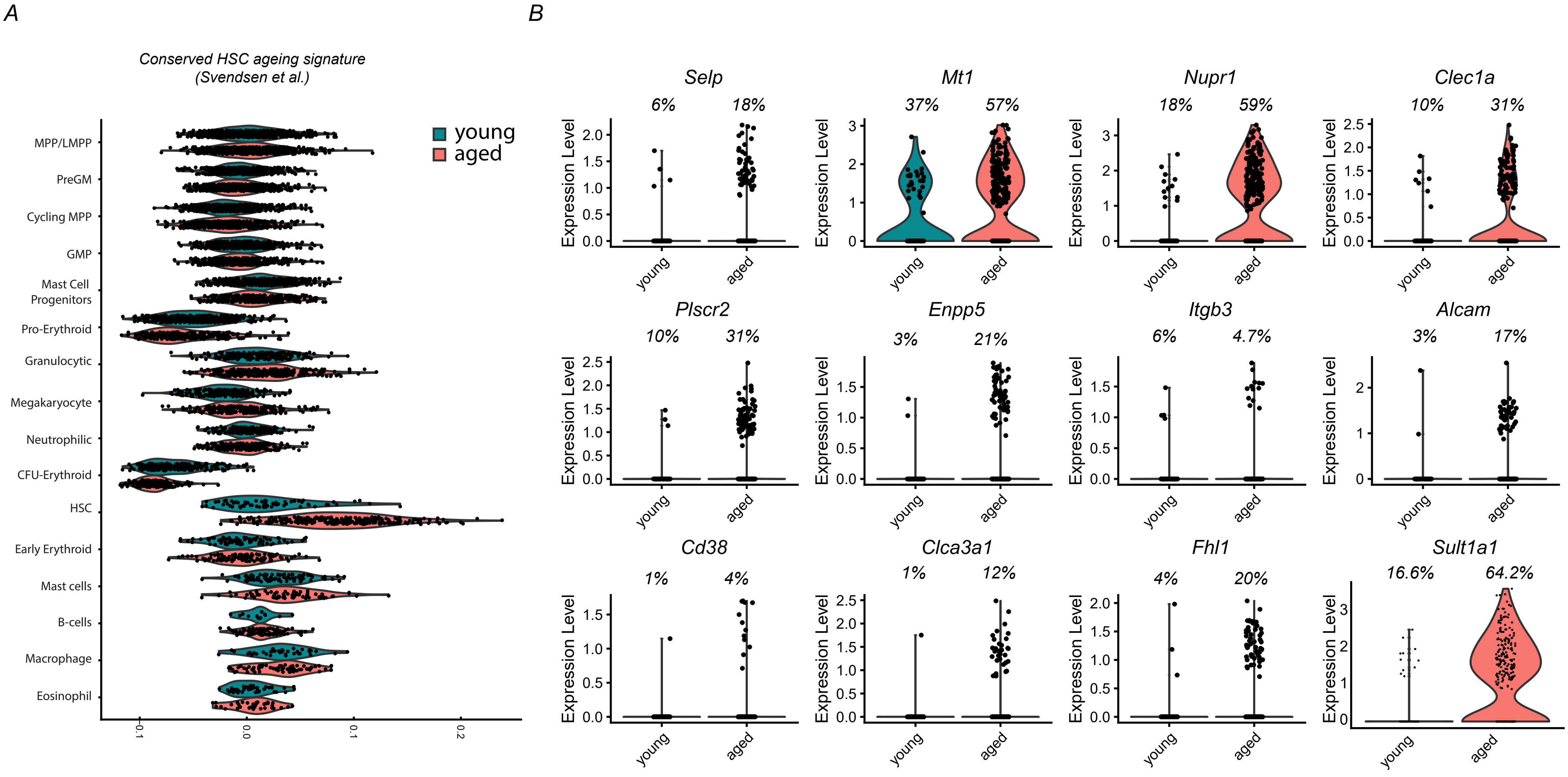

Supplementary Figure 7

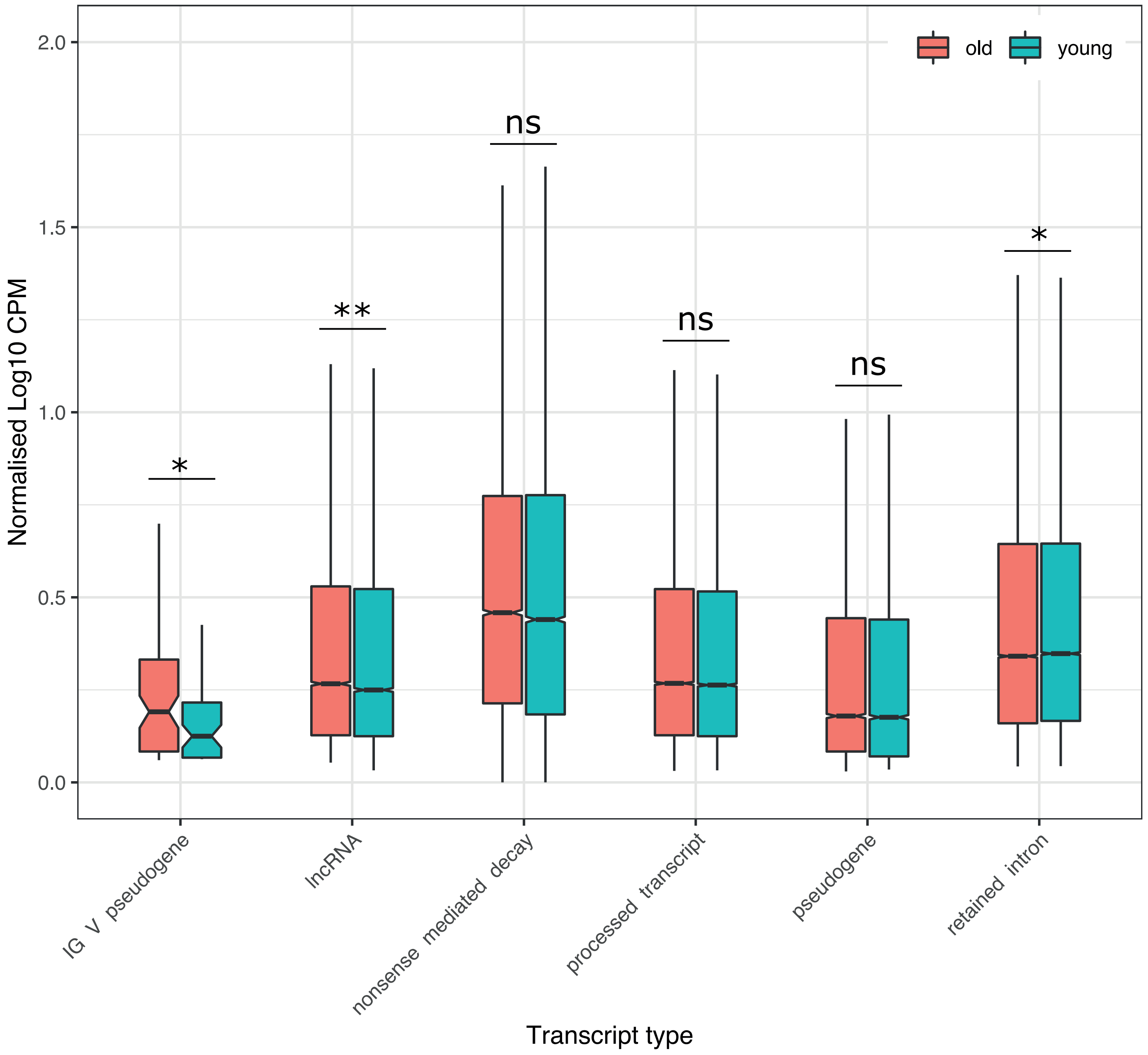

Supplementary Figure 8

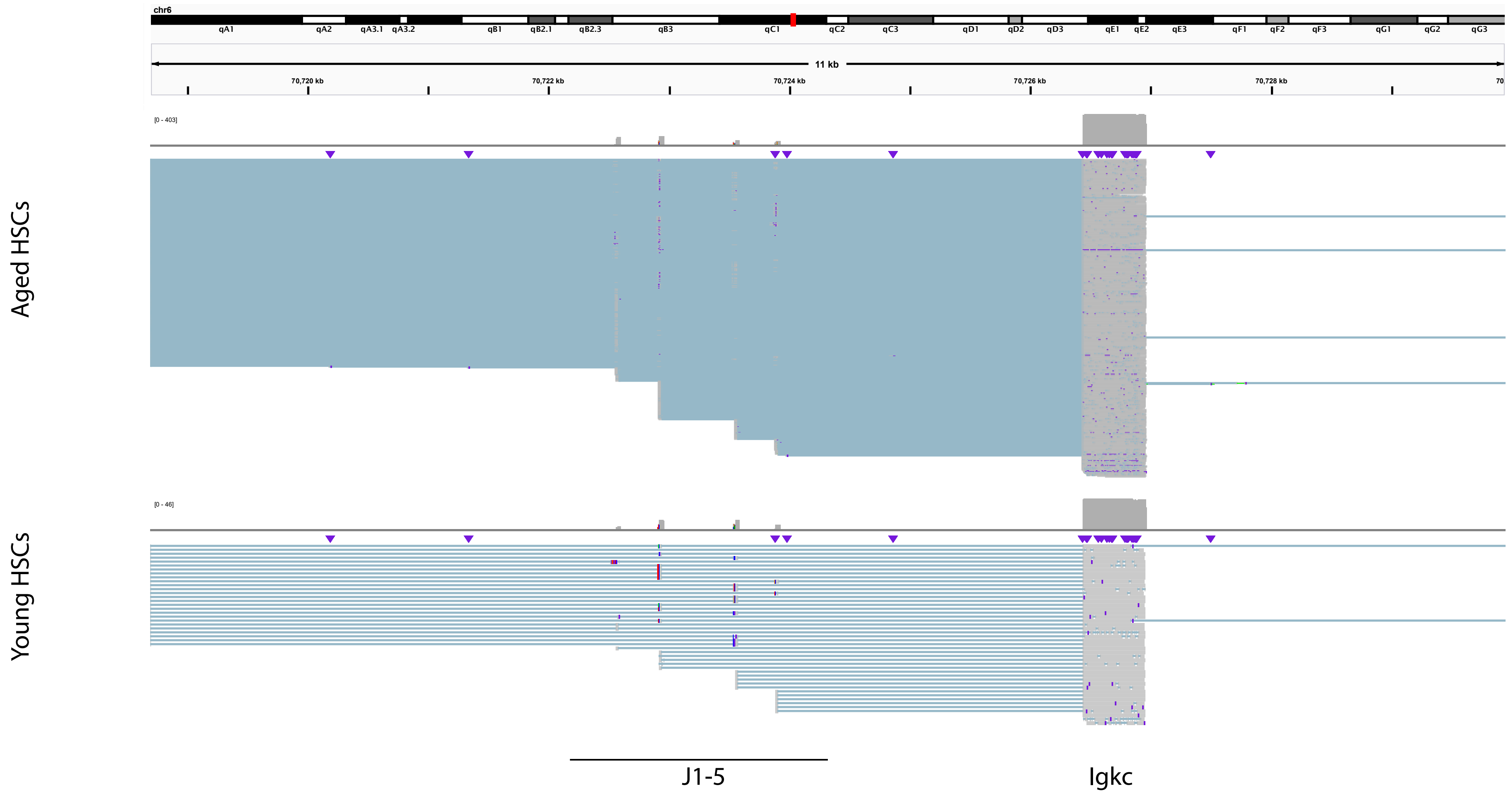

Supplementary Figure 9

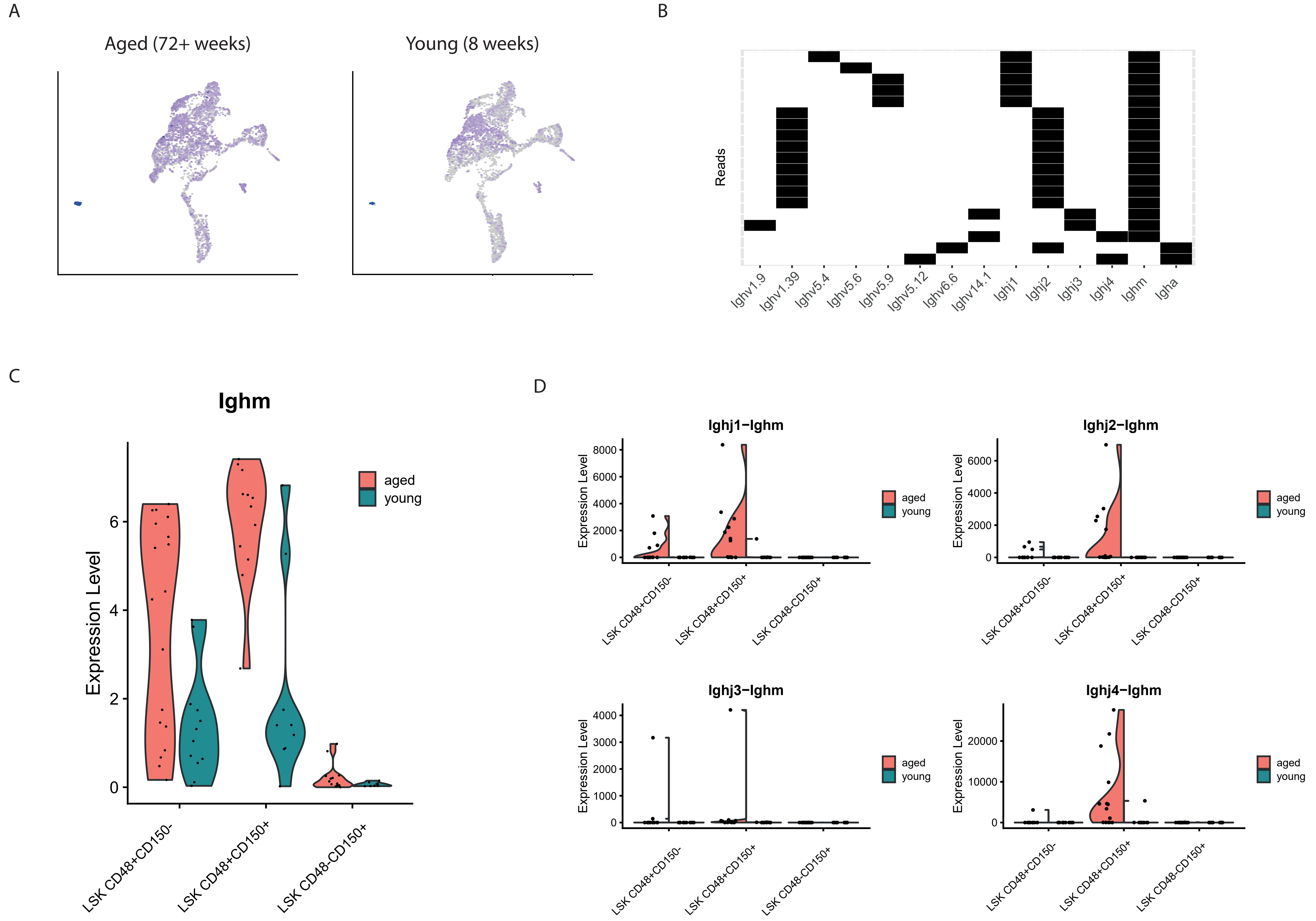

Supplementary Figure 10

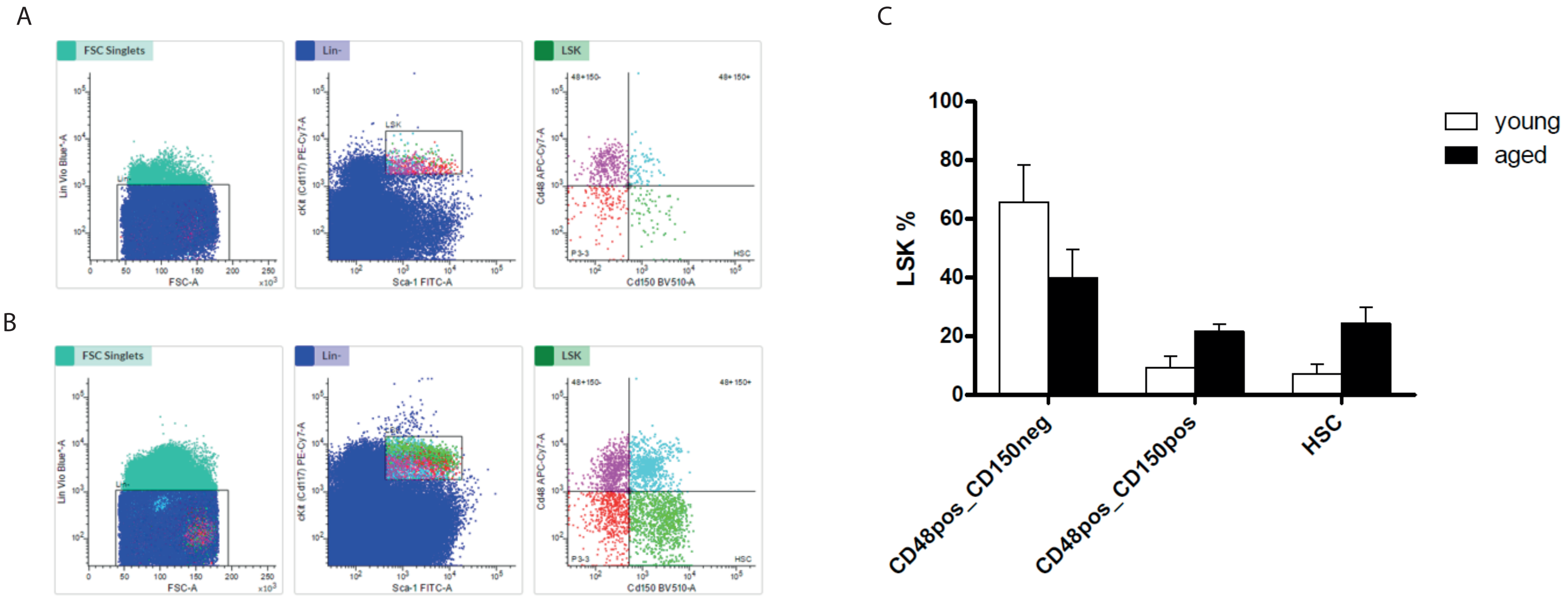

Supplementary Figure 11

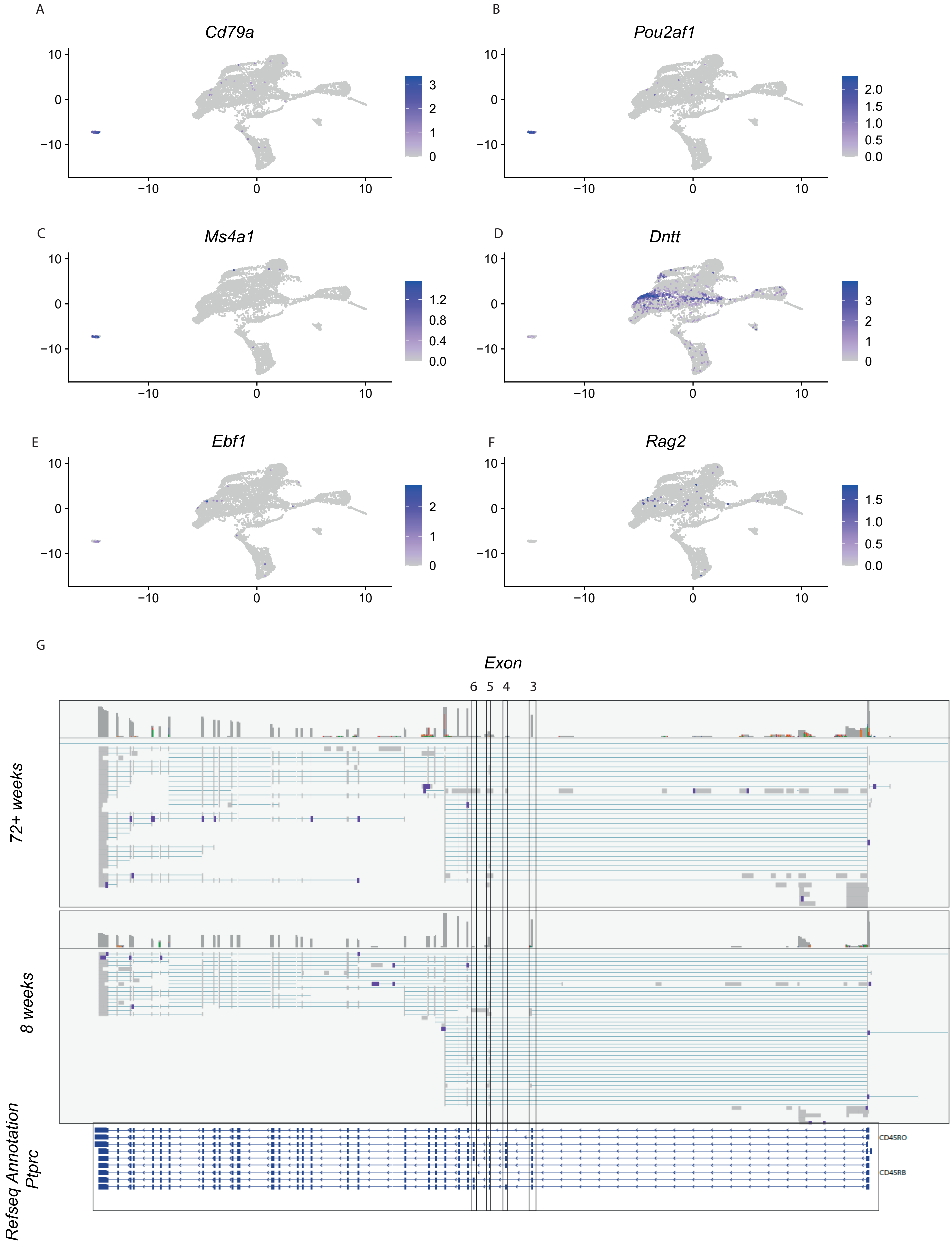

Supplementary Figure 12

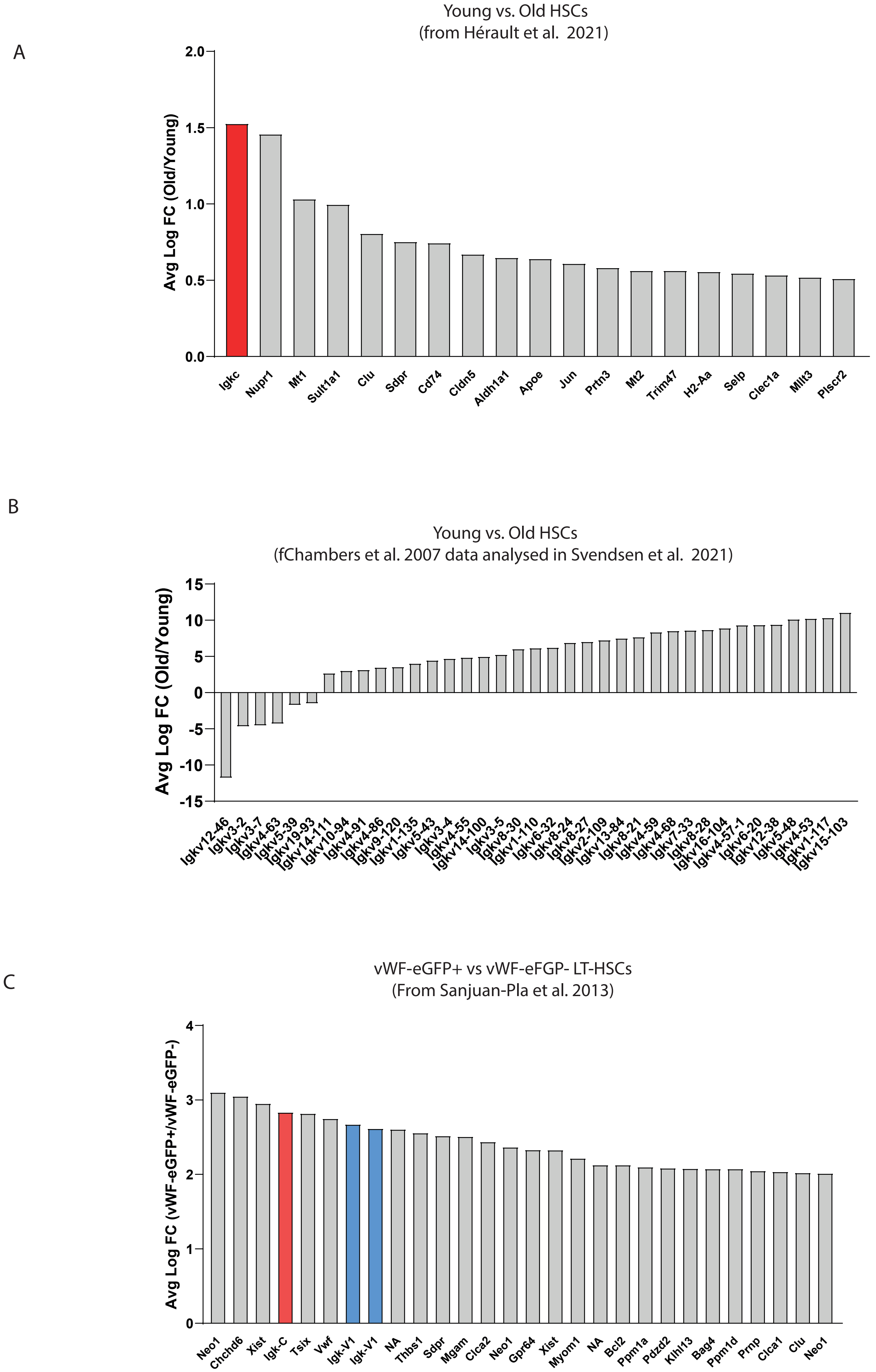

Supplementary Table 1									
Sample	1	2	3	4	5	6	Total	Percentage of Total	
Sample Type	Young	Aged	Young	Aged	Young	Aged			
Population	LK	LK	LK	LK	LK	LK			
Total CCS Reads	4071427	3388930	2541774	2145704	2970009	2785355	17903199	100	
Reads mapped to genome	4033063	3375190	2522092	2127518	2945575	2763413	17766851	99.23841544	
Reads mapped to transcriptome	3355466	2756682	1920347	1581424	2615797	2174030	14403746	80.4534765	
Reads without polyT	1188556	1043010	1036137	933035	1328830	1012957	6542525	36.54388805	
Demultiplexed Reads	1880923	1378487	876906	606000	530814	541317	5814447	32.47713998	
