# Supplementary material for: Single-cell gene and isoform expression analysis reveals signatures of ageing in haematopoietic stem and progenitor cells
Source: Commun Biol. 2023 May 24;6:558. doi: 10.1038/s42003-023-04936-6 (PMC10209181; doi:10.1038/s42003-023-04936-6)
Supplement: Supplementary file 2 — Description of Additional Supplementary Files [file 42003_2023_4936_MOESM2_ESM.pdf]

Description of Additional Supplementary Files

File name: Supplementary Data 1

Description: Marker genes for clusters (relating to Figure 1B)

File name: Supplementary Data 2

Description: Novel exons detected in long-read sequencing data.

File name: Supplementary Data 3

Description: Differential gene expression between young and aged HSCs

File name: Supplementary Data 4

Description: Source data for the graphs and charts
