# Supplementary material for: Single-cell gene and isoform expression analysis reveals signatures of ageing in haematopoietic stem and progenitor cells
Source: Commun Biol. 2023 May 24;6:558. doi: 10.1038/s42003-023-04936-6 (PMC10209181; doi:10.1038/s42003-023-04936-6)
Supplement: Supplementary file 7 — Reporting Summary [file 42003_2023_4936_MOESM7_ESM.pdf]

Corresponding author(s): Iain Macaulay, Wilfied Haerty, Laura Mincarelli

Last updated by author(s): Oct 21, 2022

Reporting Summary

Nature Portfolio wishes to improve the reproducibility of the work that we publish. This form provides structure for consistency and transparency in reporting. For further information on Nature Portfolio policies, see our Editorial Policies and the Editorial Policy Checklist.

Statistics

For all statistical analyses, confirm that the following items are present in the figure legend, table legend, main text, or Methods section.

n/a	Confirmed	
☐	☒	The exact sample size (*n*) for each experimental group/condition, given as a discrete number and unit of measurement
☐	☒	A statement on whether measurements were taken from distinct samples or whether the same sample was measured repeatedly
☐	☒	The statistical test(s) used AND whether they are one- or two-sided<br>*Only common tests should be described solely by name; describe more complex techniques in the Methods section.*
☐	☒	A description of all covariates tested
☐	☒	A description of any assumptions or corrections, such as tests of normality and adjustment for multiple comparisons
☐	☒	A full description of the statistical parameters including central tendency (e.g. means) or other basic estimates (e.g. regression coefficient) AND variation (e.g. standard deviation) or associated estimates of uncertainty (e.g. confidence intervals)
☐	☒	For null hypothesis testing, the test statistic (e.g. *F*, *t*, *r*) with confidence intervals, effect sizes, degrees of freedom and *P* value noted<br>*Give P values as exact values whenever suitable.*
☒	☐	For Bayesian analysis, information on the choice of priors and Markov chain Monte Carlo settings
☒	☐	For hierarchical and complex designs, identification of the appropriate level for tests and full reporting of outcomes
☒	☐	Estimates of effect sizes (e.g. Cohen's *d*, Pearson's *r*), indicating how they were calculated

Our web collection on statistics for biologists contains articles on many of the points above.

Software and code

Policy information about availability of computer code

Data collection	All data were collected using commercially available platforms and data processed using publicly available tools
Data analysis	All software used is publicly available and used as outlined in the methods section of the manuscript

For manuscripts utilizing custom algorithms or software that are central to the research but not yet described in published literature, software must be made available to editors and reviewers. We strongly encourage code deposition in a community repository (e.g. GitHub). See the Nature Portfolio guidelines for submitting code & software for further information.

Data

Policy information about availability of data

All manuscripts must include a data availability statement. This statement should provide the following information, where applicable:

- Accession codes, unique identifiers, or web links for publicly available datasets
- A description of any restrictions on data availability
- For clinical datasets or third party data, please ensure that the statement adheres to our policy

All data is publicly available - The sequencing data can be accessed on the NCBI-GEO archive, accession number GSE166709

Human research participants

Policy information about studies involving human research participants and Sex and Gender in Research.

Reporting on sex and gender	n/a
Population characteristics	n/a
Recruitment	n/a
Ethics oversight	n/a

Note that full information on the approval of the study protocol must also be provided in the manuscript.

Field-specific reporting

Please select the one below that is the best fit for your research. If you are not sure, read the appropriate sections before making your selection.

☒ Life sciences ☐ Behavioural & social sciences ☐ Ecological, evolutionary & environmental sciences

For a reference copy of the document with all sections, see nature.com/documents/nr-reporting-summary-flat.pdf

Life sciences study design

All studies must disclose on these points even when the disclosure is negative.

Sample size	for scRNA-seq - 3 samples (minimum) per condition
Data exclusions	scRNA-seq data from cells with low mapping rates or high mitochondrial gene counts were excluded
Replication	finding on single-cell splicing from long read data were validated with qPCR or orthogonal approaches for single-cell sequencing
Randomization	n/a
Blinding	n/a

Reporting for specific materials, systems and methods

We require information from authors about some types of materials, experimental systems and methods used in many studies. Here, indicate whether each material, system or method listed is relevant to your study. If you are not sure if a list item applies to your research, read the appropriate section before selecting a response.

Materials & experimental systems

n/a	Involved in the study	
☐	☒	Antibodies
☒	☐	Eukaryotic cell lines
☒	☐	Palaeontology and archaeology
☐	☒	Animals and other organisms
☒	☐	Clinical data
☒	☐	Dual use research of concern

Methods

n/a	Involved in the study	
☒	☐	ChIP-seq
☐	☒	Flow cytometry
☒	☐	MRI-based neuroimaging

Antibodies

Antibodies used	Cd105-PE, clone MJ7/18, Miltenyi; Cd4-Vioblue, clone REA604, Miltenyi; Cd11b-Vioblue, clone REA592, Miltenyi; Cd117-Pe Vio770, clone REA791, Miltenyi; Cd8a-Vioblue, clone 53-6.7, Miltenyi; CD50-Vioblue, clone REA421, Miltenyi; CD45R-Vioblue, clone REA755, Miltenyi; GR1-Vioblue, clone REA810, Miltenyi; Sca-APC, clone REA422, Miltenyi; CD48-APC Cy7, clone HM48-1, Miltenyi; CD150-BV510, clone TC15-12F12, CD34-PeCy5, MEC147, Miltenyi
Validation	all antibodies are commercially available and validated

Animals and other research organisms

Policy information about studies involving animals; ARRIVE guidelines recommended for reporting animal research, and Sex and Gender in Research

Laboratory animals	C57BL/6
Wild animals	n/a
Reporting on sex	all samples were from female mice
Field-collected samples	n/a
Ethics oversight	UK Home Office

Note that full information on the approval of the study protocol must also be provided in the manuscript.

Flow Cytometry

Plots

Confirm that:

☒ The axis labels state the marker and fluorochrome used (e.g. CD4-FITC).

☒ The axis scales are clearly visible. Include numbers along axes only for bottom left plot of group (a 'group' is an analysis of identical markers).

☒ All plots are contour plots with outliers or pseudocolor plots.

☒ A numerical value for number of cells or percentage (with statistics) is provided.

Methodology

Sample preparation	Samples were prepared from crushed bone marrow, lineage depleted and stained with the appropriate antibody cocktail.
Instrument	BD FACSMelody
Software	Chorus
Cell population abundance	purity was assessed by sequencing of the sorted populations, in addition to backgating and post-sort purity testing.
Gating strategy	Lin- cKit+ cells were sorting for 10X scRNA-seq. Lin- Sca-1+ cKit+ CD150+/- CD48+/- cells were sorted for single HSC and low input HSC sequencing experiments.

☒ Tick this box to confirm that a figure exemplifying the gating strategy is provided in the Supplementary Information.
